# Identification of Fungicidal 2,6-Disubstituted Quinolines with Activity against *Candida* Biofilms

**DOI:** 10.3390/molecules171012243

**Published:** 2012-10-18

**Authors:** Nicolas Delattin, Dorothée Bardiot, Arnaud Marchand, Patrick Chaltin, Katrijn De Brucker, Bruno P. A. Cammue, Karin Thevissen

**Affiliations:** 1 Centre of Microbial and Plant Genetics, Katholieke Universiteit Leuven, Kasteelpark Arenberg 20, B-3001, Heverlee, Belgium; 2 CISTIM Leuven vzw, Minderbroedersstraat 12, B-3000, Leuven, Belgium; 3 Centre for Drug Design and Discovery, Minderbroedersstraat 8a, B-3000, Leuven, Belgium

**Keywords:** antifungal agents, *Candida spp.*, 2,6-disubstituted quinolines, reactive oxygen species, structure-activity relationships

## Abstract

We have identified two subseries of 2,6-disubstituted quinolines, consisting of 6-amide and 6-urea derivatives, which are characterized by fungicidal activity against *Candida albicans* with minimal fungicidal concentration (MFC) values < 15 µM. The 6-amide derivatives displayed the highest fungicidal activity against *C. albicans*, in particular compounds **1**, **5** and **6** characterized by MFC values of 6.25–12.5 µM. Compounds **1** and **5** of this series displayed fungicidal activity against the emerging pathogen *Candida glabrata*(MFC < 50 µM). The 6-amide derivatives **1**, **2**, **5**, and **6** and the 6-urea derivatives **10**, **12**, **13** and **15** could also eradicate *C. albicans* biofilms. We found that the 6-urea derivatives **10**, **13**, and **15** induced accumulation of endogenous reactive oxygen species in *Candida albicans* biofilms.

## 1. Introduction

Pathogenic yeasts from the genus *Candida* can cause serious infections in humans, particularly in immunocompromised patients and are now recognized as the major agents of hospital acquired (nosocomial) infections. *Candida* is recognized as the fourth most common cause of bloodstream infections in the United States, with a high attributable mortality rate [1]. While *Candida albicans* remains the most common pathogen, non-*albicans* *Candida* species, including *C. glabrata* and *C. krusei*, with greater resistance to triazoles are being increasingly isolated [1].

Apart from their existence as free-living or planktonic forms, various yeast and fungal species are known to form biofilms upon contact with various surfaces. Moreover, biofilm formation is thought to be a critical step in the development of clinical infections in general [2]. Biofilms consist of dense layers of microorganisms that are surrounded by a self-produced extracellular polymer matrix, and are resistant to most of the currently used antibiotics [3]. Fungal biofilms, especially those of the pathogen *C. albicans*, are a cause of infections associated with medical devices like indwelling intravascular catheters [4]. Hence, antifungal compounds that can eradicate *Candida* biofilms are of great therapeutic potential.

The increase of fungal resistance to standard treatments [5,6] has stimulated the search for new antifungal drugs. When designing or screening for novel antifungal drugs, fungicidal activity is generally preferred over fungistatic activity, since it pinpoints to inhibition of targets that are essential for fungal growth [7] or induction of an active cell death pathway, such as apoptosis [8]. An important asset for a novel antifungal lead compound is its potential activity against fungal biofilms.

This study describes the identification and characterization of a series of fungicidal 2,6-disubstituted quinoline derivatives with antibiofilm activity. Various antifungal compounds that are active against *Candida* biofilms, like miconazole [9] and amphotericin B [10], induce an increased accumulation of endogenous reactive oxygen species (ROS) in biofilm cells. Therefore, we assessed whether these novel antibiofilm compounds also lead to an increased accumulation of endogenous ROS in *C. albicans* biofilms.

Apart from the observed activity of quinolines against fungal biofilms stated in this study, following reports document the activity of quinolines for other therapeutic indications. Lanza and coworkers identified substituted 4,6-diaminoquinolines as inhibitors of C5a receptor binding, a protein involved in inflammatory diseases [11]. Furthermore, aminoquinolines are known as antimalarial drugs [12] and chloroquin, a 4-aminoquinoline was shown to exhibit antifungal activity against the opportunistic fungus *Penicillium marneffei* [13]. Finally, 6-acylamino-2-aminoquinolines were identified as potent Melanin-Concentrating Hormone 1 Receptor (MCH1R) antagonists, a potential target to tackle obesity [14]. 

## 2. Results and Discussion

### 2.1. Screening and Hit Identification

A screening campaign for fungicidal compounds resulted in the identification of a fungicidal 2,6-disubstituted quinoline derivative, namely 4-*tert*-butyl-*N*-(4-methyl-2-(4-methylpiperazin-1-yl)quinolin-6-yl)benzamide (**9**, Table 1a). This screening campaign, which previously led to the identification of antifungal carbazoles [15], consisted of a two-step procedure. In a first step, approximately 5,000 compounds representing diverse chemical classes were screened for antifungal activity against *C. albicans*, in an antifungal spot assay on *C. albicans*-inoculated agar using a single compound dose (100 µg/mL). Next, fungicidal activity of antifungal hits was assessed using two-fold dilution series of the compounds, allowing determination of their minimal fungicidal concentration (MFC) against *C. albicans* in phosphate buffered saline (PBS). The MFC was defined as the minimal compound concentration that results in 99% cell death of the inoculum as compared to the DMSO control treatment [15]. Compound **9**, together with the antifungal carbazoles [15], was among the most potent fungicidal compounds identified in this screening campaign. This compound was characterized by fungicidal activity against *C. albicans* with a MFC value of 50 µM (Table 2).

**Table 1:** Selected 6-amide (**a**) and 6-urea (**b**) derivatives of 2,6-disubstituted quinolines.

(**a**) 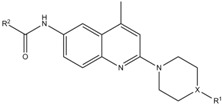			
**Compound**	**R^1^**	**X**	**R^2^**
**1**	ethyl	N	4-*tert*-butylphenyl
**2**	ethyl	N	4-butylphenyl
**3**	2-pyrimidyl	N	4-*tert*-butylphenyl
**4**	methyl	CH	4-*tert*-butylphenyl
**5**	methyl	N	4-butylphenyl
**6**	methyl	N	4-*tert*-butylcyclohexyl
**7**	methyl	N	5-chloro-2-methoxyphenyl
**8**	methyl	N	2,4-dimethoxyphenyl
**9**	methyl	N	4-*tert*-butylphenyl
(**b**) 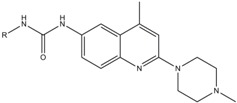			
**Compound**		**R**	
**10**		3-chlorophenyl	
**11**		phenyl	
**12**		4-chlorophenyl	
**13**		3,5-dimethylphenyl	
**14**		3,4-dimethylphenyl	
**15**		4-ethylphenyl	
**16**		2-methylphenyl	
**17**		2-chlorophenyl	
**18**		4-bromophenyl	

### 2.2. Structure-Activity Relationship

To analyze the structural determinants for fungicidal activity against *C. albicans* of this new class of compounds, we assessed the MFC of 18 commercially available derivatives divided into two subseries of 2,6-disubstituted quinolines, consisting of 6-amide derivatives (Table 1a) and 6-urea derivatives (Table 1b), and of the reference antifungals amphotericin B (AmB) and miconazole (Mico) (Table 2). Next, we assessed their fungicidal activity against *C. glabrata*, as well as their potential antibiofilm activity in terms of the biofilm eradicating concentration 50 (BEC_50_), which is the minimal concentration of compound resulting in 50% killing of the *C. albicans* biofilm cells.

**Table 2:** Antifungal activity of 2,6-disubstituted quinolines against *C. albicans*, *C. glabrata* and *C. albicans* biofilms.

Compound	*C. albicans* MFC ^[a]^ (µM)	*C. glabrata* MFC ^[a]^ (µM)	*C. albicans* BEC_50_^[b]^ (µM)
**1**	12.5	25	25
**2**	25	>50	25
**3**	>50	>50	>50
**4**	>50	>50	>50
**5**	6.25	25	25
**6**	12.5	>50	25
**7**	50	>50	>50
**8**	50	>50	>50
**9**	50	>50	>50
**10**	50	>50	25
**11**	>50	>50	>50
**12**	>50	>50	50
**13**	>50	>50	50
**14**	50	>50	>50
**15**	>50	>50	50
**16**	>50	>50	>50
**17**	25	>50	>50
**18**	>50	>50	>50
**AmB**	<3.25	<12.5	<12.5
**Mico**	>50	>50	25

^[a]^ Minimal fungicidal concentration; ^[b]^ minimal concentration that results in 50% killing of biofilm cells.

Compounds **1**, **2**, **5**, **6** and **17** were the most potent fungicidal derivatives against *C. albicans* (MFC = 6.25–25 µM). Together with compound **10**, these compounds (except for **17**) were able to eradicate *C. albicans* biofilms with a BEC_50_ value identical to the one obtained with miconazole (BEC_50_ = 25 µM), whereas compounds **12**, **13** and **15** showed moderate antibiofilm activity (BEC_50_ = 50 µM). Only compounds **1** and **5** showed fungicidal activity against the emerging pathogen *C. glabrata* (MFC = 25 µM). The early SAR study showed that the antifungal activity against *C. albicans* was partially driven by the presence of a piperazine moiety on the 2 position of the quinoline scaffold, since compound **9** showed a modest MFC value (50 µM) whereas compound **4** (piperidine moiety) did not exhibit any fungicidal activity at a concentration higher than 50 µM. In addition, the introduction of an aromatic (pyrimidyl) substituent on the piperazine moiety (compound **3**) instead of a small methyl group (compound **1**) led to a complete loss of both antifungal (*C. albicans* and *C. glabrata*) and antibiofilm activities. Inversely, antifungal (*C. albicans*) as well as antibiofilm activities were improved by changing the phenyl ring (compound **9**) into a cyclohexyl ring (compound **6**). The urea derivatives (Table 1b) were in general not active or less potent than the corresponding amide derivatives (Table 1a). Only compounds **10** and **17** showed some antibiofilm (BEC_50_ = 25 µM) or antifungal activity (*C. albicans* MFC = 25 µM), respectively. Note that the above SAR was only based on biological data. We did not use QSAR or another computational algorithm.

### 2.3. Accumulation of Endogenous ROS

**Figure 1:**
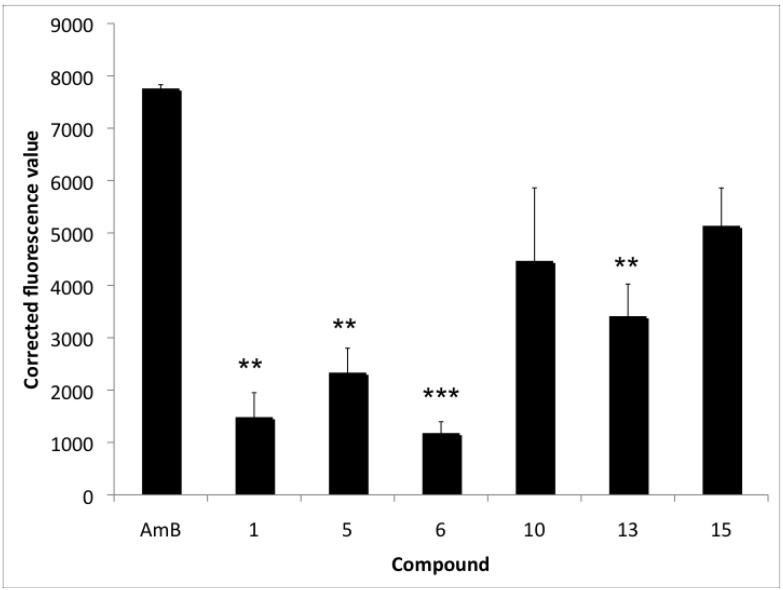
Endogenous ROS induction in *C. albicans* biofilm cells by the most potent compounds against *C. albicans* biofilms. Corrected fluorescence values (CFV) of ROS accumulation by 16 h old *C. albicans* biofilm cells after treatment (24 h) with 50 μM of the compounds in PBS with a final DMSO background of 2.5%. Experiments were performed in duplicate and repeated twice. ** *p* < 0.01; *** *p* < 0.001.

Antifungal compounds that are active against *C. albicans* biofilms, like miconazole and amphotericin B, were previously shown to induce an increased accumulation of ROS in biofilm cells [9,10] and planktonic cells [16,17,18]. To assess whether the fungicidal 2,6-disubstituted quinoline derivatives were also able to induce ROS, we measured the endogenous ROS levels via the fluorescent dye 2′,7′-dichlorodihydrofluorescein diacetate (H2DCFDA), after induction by the most potent derivatives on *C. albicans* planktonic and biofilm cells. The most active fungicidal derivatives (compounds **1**, **2**, **5** and **6**) against planktonic *C. albicans* cells, *i.e.*, with MFC values ranging between 6.25–25 µM did not induce ROS accumulation in planktonic cells upon 1, 3 or 5 h of treatment (data not shown). In contrast, *C. albicans* biofilm cells treated with the most active antibiofilm compounds (compounds **1**, **5**, **6**, **10**, **13** and **15**), *i.e.*, compounds characterized with BEC_50_ values ≤ 50 µM, induced endogenous ROS accumulation on biofilm cells after 24 h incubation (Figure 1). Compounds **10**, **13** and **15** were characterized by a high capacity to induce ROS accumulation in biofilm cells as indicated by corrected fluorescence values (CFV) > 3,000. Compounds **10** and **15** can induce ROS to the same extent as AmB (*p* > 0.05). CFV are the fluorescence values of the biofilm cells treated with the antifungal compounds subtracted with the value of the DMSO control. Compounds **1**, **5** and **6** were characterized by an intermediate ROS-induction capacity in biofilm cells (1,000 < CFV < 3,000).

## 3. Experimental

### 3.1. Chemical Compounds

All compounds: 4-*tert*-butyl-*N*-(2-(4-ethylpiperazin-1-yl)-4-methylquinolin-6-yl)benzamide (**1**); 4-butyl-*N*-(2-(4-ethylpiperazin-1-yl)-4-methylquinolin-6-yl)benzamide (**2**); 4-*tert*-butyl-*N*-(4-methyl-2-(4-(pyrimidin-2-yl)piperazin-1-yl)quinolin-6-yl)benzamide (**3**); 4-*tert*-butyl-*N*-(4-methyl-2-(4-methyl- piperidin-1-yl)quinolin-6-yl)benzamide (**4**); 4-butyl-*N*-(4-methyl-2-(4-methylpiperazin-1-yl)quinolin-6-yl)benzamide (**5**); 4-*tert*-butyl-*N*-(4-methyl-2-(4-methylpiperazin-1-yl)quinolin-6-yl)cyclohexane-carboxamide (**6**); 5-chloro-2-methoxy-*N*-(4-methyl-2-(4-methylpiperazin-1-yl)quinolin-6-yl)benz-amide (**7**); 2,4-dimethoxy-*N*-(4-methyl-2-(4-methylpiperazin-1-yl)quinolin-6-yl)benzamide (**8**); 4-*tert*-butyl-*N*-(4-methyl-2-(4-methylpiperazin-1-yl)quinolin-6-yl)benzamide (**9**); 1-(3-chlorophenyl)-3-(4-methyl-2-(4-methylpiperazin-1-yl)quinolin-6-yl)urea (**10**); 1-(4-methyl-2-(4-methylpiperazin-1-yl)-quinolin-6-yl)-3-phenylurea (**11**); 1-(4-chlorophenyl)-3-(4-methyl-2-(4-methyl-piperazin-1-yl)quinol-in-6-yl)urea (**12**); 1-(3,5-dimethylphenyl)-3-(4-methyl-2-(4-methylpiperazin-1-yl)quinolin-6-yl)urea (**13**); 1-(3,4-dimethylphenyl)-3-(4-methyl-2-(4-methylpiperazin-1-yl)quinolin-6-yl)urea (**14**); 1-(4-ethylphenyl)-3-(4-methyl-2-(4-methylpiperazin-1-yl)quinolin-6-yl)urea (**15**); 1-(4-methyl-2-(4-methyl-piperazin-1-yl)quinolin-6-yl)-3-o-tolylurea (**16**); 1-(2-chlorophenyl)-3-(4-methyl-2-(4-methylpiperazin-1-yl)quinolin-6-yl)urea (**17**); 1-(4-bromophenyl)-3-(4-methyl-2-(4-methylpiperazin-1-yl)quinolin-6-yl)-urea (**18**) were purchased from ChemDiv (San Diego, CA, USA). Synthesis of the amide derivatives has been described previously [14].

### 3.2. Strains and Media

The yeast strains used in this study were *Candida albicans* strain SC5314 [19] and *Candida glabrata* strain BG2 [20]. Overnight cultures were grown in YPD (1% yeast extract, 2% peptone and 2% glucose). Biofilms were grown in SC medium (0.8 g/L CSM, complete amino acid supplement mixture, Bio 101 Systems; 6.5 g/L YNB, yeast nitrogen base; 20 g/L glucose). PBS consists of 8 g/L NaCl, 0.2 g/L KCL, 1.44 g/L Na_2_HPO_4_ and 0.24 g/L KH_2_PO_4_ (pH 7.4). Reference antimycotics, amphotericin B and miconazole were purchased from Sigma-Aldrich (St. Louis, MO, USA). 

### 3.3. Fungicidal Activity

The fungicidal activity of antifungal compounds against *C. albicans* and *C. glabrata* was determined in PBS and the MFC for each compound was calculated according to the definition of Thevissen and coworkers [15]. To this end, overnight cultures of *C. albicans* or *C. glabrata* in YPD were 1/200 and 1/400 diluted in PBS, respectively, and treated with the compounds or DMSO (2.5% as solvent control) for 24 h at 37 °C. After 24 h, the MFC was calculated by counting the number of colony forming units (CFUs) as described previously [21].

### 3.4. Antibiofilm Activity

The activity of the compounds against 16 h-old *C. albicans* SC5314 biofilms was determined in PBS and the BEC_50_ for each compound was calculated. *C. albicans* biofilms were grown in sterile 96-well microtiter plates (TPP, Trasadingen, Switzerland). Overnight cultures of *C. albicans* in YPD were washed in milliQ water and 1/20 diluted in fresh SC medium. 100 µL of this culture was added to each well of a round-bottomed polystyrene 96-well microtiter plate. Following 1 h of adhesion at 37 °C, the supernatant was removed, the wells were rinsed using milliQ water and incubated with 100 µL of fresh SC medium for a growth phase of 16 h. After 16 h of biofilm formation at 37 °C, wells were rinced with PBS and incubated with 100 µL of the antifungal compounds or DMSO (2.5%) in PBS at 37 °C. After 24 h of incubation, biofilms were washed and resuspended in PBS by vigorous vortexing. The BEC_50_ was determined by counting the number of colony forming units (CFU) as described previously [21].

### 3.5. Measurement of ROS Accumulation

Endogenous amounts of ROS, after treatment of planktonic and biofilm cells with the compounds (2.5% DMSO) were measured by a fluorometric assay with 2′,7′-dichlorodihydrofluorescein diacetate (H2DCFDA; Molecular Probes, Inc., Eugene, OR, USA) [18,22]. Fluorescence was measured using a fluorescence spectrometer as described [18] and fluorescence values of the samples were corrected by subtracting the fluorescence values of the antifungal compounds with the value of the DMSO control.

## 4. Conclusions

In this study we identified a new series of fungicidal 2,6-disubstituted quinoline derivatives. Among these derivatives, compounds **1**, **2**, **5**, **6** and **17** were characterized by the highest fungicidal activity against planktonic cultures of *C. albicans*. Moreover compounds **1** and **5** also showed activity against the emerging pathogen *C. glabrata*. Furthermore, compounds **1**, **2**, **5**, **6** and **10** showed the most potent activity against *C. albicans* biofilms, with BEC_50_ values comparable to the one obtained with miconazole (BEC_50_ = 25 µM). There are several reports documenting the involvement of ROS in the fungicidal activity of compounds against *C. albicans* biofilms, as in case of miconazole and amphotericin B [9,10]. Compounds **10**, **13** and **15** showed a high capacity to induce ROS accumulation in *C. albicans* biofilm cells. Whether the induction of ROS accumulation is the main mechanism of the antibiofilm activity of these new compounds still needs to be investigated. To our knowledge this is the first report regarding the fungicidal activity of these 2,6-disubstituted quinoline derivatives. 

## References

[1] Marr K.A. (2004). Invasive Candida infections: The changing epidemiology. Oncology (Williston Park).

[2] Fanning S., Mitchell A.P. (2012). Fungal biofilms. PLoS Pathog..

[3] Ramage G., Mowat E., Jones B., Williams C., Lopez-Ribot J. (2009). Our current understanding of fungal biofilms. Crit. Rev. Microbiol..

[4] Kuhn D.M., Ghannoum M.A. (2004). Candida biofilms: Antifungal resistance and emerging therapeutic options. Curr. Opin. Investig. Drugs.

[5] Klepser M.E. (2006). Candida resistance and its clinical relevance. Pharmacotherapy.

[6] Chamilos G., Kontoyiannis D.P. (2005). Update on antifungal drug resistance mechanisms of Aspergillus fumigatus. Drug Resist. Updat..

[7] Odds F.C. (2005). Genomics, molecular targets and the discovery of antifungal drugs. Rev. Iberoam. Micol..

[8] Thevissen K., Hillaert U., Meert E.M., Chow K.K., Cammue B.P., Van Calenbergh S., Francois I.E. (2008). Fungicidal activity of truncated analogues of dihydrosphingosine. Bioorg. Med. Chem. Lett..

[9] Vandenbosch D., Braeckmans K., Nelis H.J., Coenye T. (2010). Fungicidal activity of miconazole against Candida spp. biofilms. J. Antimicrob. Chemother..

[10] Al-Dhaheri R.S., Douglas L.J. (2010). Apoptosis in Candida biofilms exposed to amphotericin B. J. Med. Microbiol..

[11] Lanza T.J., Durette P.L., Rollins T., Siciliano S., Cianciarulo D.N., Kobayashi S.V., Caldwell C.G., Springer M.S., Hagmann W.K. (1992). Substituted 4,6-diaminoquinolines as inhibitors of C5a receptor binding. J. Med. Chem..

[12] Foley M., Tilley L. (1998). Quinoline antimalarials: mechanisms of action and resistance and prospects for new agents. Pharmacol. Ther..

[13] Taramelli D., Tognazioli C., Ravagnani F., Leopardi O., Giannulis G., Boelaert J.R. (2001). Inhibition of intramacrophage growth of Penicillium marneffei by 4-aminoquinolines. Antimicrob. Agents Chemother..

[14] Ulven T., Frimurer T.M., Receveur J.M., Little P.B., Rist O., Norregaard P.K., Hogberg T. (2005). 6-Acylamino-2-aminoquinolines as potent melanin-concentrating hormone 1 receptor antagonists. Identification, structure-activity relationship, and investigation of binding mode. J. Med. Chem..

[15] Thevissen K., Marchand A., Chaltin P., Meert E.M., Cammue B.P. (2009). Antifungal carbazoles. Curr. Med. Chem..

[16] Kobayashi D., Kondo K., Uehara N., Otokozawa S., Tsuji N., Yagihashi A., Watanabe N. (2002). Endogenous reactive oxygen species is an important mediator of miconazole antifungal effect. Antimicrob. Agents Chemother..

[17] Thevissen K., Ayscough K.R., Aerts A.M., Du W., De Brucker K., Meert E.M., Ausma J., Borgers M., Cammue B.P., Francois I.E. (2007). Miconazole induces changes in actin cytoskeleton prior to reactive oxygen species induction in yeast. J. Biol. Chem..

[18] Francois I.E., Thevissen K., Pellens K., Meert E.M., Heeres J., Freyne E., Coesemans E., Viellevoye M., Deroose F., Martinez Gonzalez S. (2009). Design and synthesis of a series of piperazine-1-carboxamidine derivatives with antifungal activity resulting from accumulation of endogenous reactive oxygen species. ChemMedChem.

[19] Fonzi W.A., Irwin M.Y. (1993). Isogenic strain construction and gene mapping in Candida albicans. Genetics.

[20] Kaur R., Ma B., Cormack B.P. (2007). A family of glycosylphosphatidylinositol-linked aspartyl proteases is required for virulence of Candida glabrata. Proc. Natl. Acad. Sci. USA.

[21] Aerts A.M., Carmona-Gutierrez D., Lefevre S., Govaert G., Francois I.E., Madeo F., Santos R., Cammue B.P., Thevissen K. (2009). The antifungal plant defensin RsAFP2 from radish induces apoptosis in a metacaspase independent way in Candida albicans. FEBS Lett..

[22] Bink A., Vandenbosch D., Coenye T., Nelis H., Cammue B.P., Thevissen K. (2011). Superoxide dismutases are involved in Candida albicans biofilm persistence against miconazole. Antimicrob. Agents Chemother..

